# Astrocyte and Oligodendrocyte Cross-Talk in the Central Nervous System

**DOI:** 10.3390/cells9030600

**Published:** 2020-03-03

**Authors:** Erik Nutma, Démi van Gent, Sandra Amor, Laura A. N. Peferoen

**Affiliations:** 1 Department of Pathology, Amsterdam UMC, Location VUmc, 1081 HV Amsterdam, The Netherlands; 2 Centre for Neuroscience and Trauma, Blizard Institute, Barts and the London School of Medicine & Dentistry, Queen Mary University of London, London E1 2AT, UK

**Keywords:** astrocytes, oligodendrocytes, white matter disease, cross-talk, CNS, glial cells.

## Abstract

Over the last decade knowledge of the role of astrocytes in central nervous system (CNS) neuroinflammatory diseases has changed dramatically. Rather than playing a merely passive role in response to damage it is clear that astrocytes actively maintain CNS homeostasis by influencing pH, ion and water balance, the plasticity of neurotransmitters and synapses, cerebral blood flow, and are important immune cells. During disease astrocytes become reactive and hypertrophic, a response that was long considered to be pathogenic. However, recent studies reveal that astrocytes also have a strong tissue regenerative role. Whilst most astrocyte research focuses on modulating neuronal function and synaptic transmission little is known about the cross-talk between astrocytes and oligodendrocytes, the myelinating cells of the CNS. This communication occurs via direct cell-cell contact as well as via secreted cytokines, chemokines, exosomes, and signalling molecules. Additionally, this cross-talk is important for glial development, triggering disease onset and progression, as well as stimulating regeneration and repair. Its critical role in homeostasis is most evident when this communication fails. Here, we review emerging evidence of astrocyte-oligodendrocyte communication in health and disease. Understanding the pathways involved in this cross-talk will reveal important insights into the pathogenesis and treatment of CNS diseases.

## 1. Introduction

Astrocytes, the most abundant glial cell type in the central nervous system (CNS), have long been considered to be cells that only respond to damage in CNS diseases. This view is gradually changing with the accumulating evidence that astrocytes fulfil many functions in health, during development and in response to damage [1]. Astrocytes regulate processes critical for cell-cell interactions and homeostasis such as ion and water transport, pH, neuroplasticity, synapse pruning and cerebral blood flow thus providing trophic and metabolic support to all cells in the CNS. Astrocytes also play a major role in maintaining the blood-brain barrier (BBB) and blood-cerebrospinal fluid barrier. During CNS injury, infection and inflammation astrocytes produce a wide range of pro-inflammatory factors including chemokines, cytokines, increased expression of innate immune receptors and molecules including MHC-II [2,3,4,5]. On the other hand, astrocytes produce anti-inflammatory cytokines, heat shock proteins and neuroprotective factors aiding in processes such as neuroregeneration and remyelination [2]. These different characteristics present the astrocyte as a versatile player in regulatory processes depending on context and time of injury and disease. While much of the knowledge of astrocytes relates to their interaction with neurons and neuronal functions astrocytes collaborate and impact on other cells within the CNS as well, such as endothelial cells and pericytes in BBB formation. They also share their lineage with oligodendrocytes and interact with these myelin forming cells by sharing gap junctions allowing passage of small metabolites and molecules for communication [6].

Oligodendrocytes have the highest metabolic rate of cells in the CNS, producing myelin up to three times their weight per day for up to 50 axons each. The myelin sheaths are critical for action potentials and need to be maintained constantly [7]. Additionally, oligodendrocytes provide axons with trophic support and are crucial for neuronal functionality [2,7]. Due to their high turnover of myelin oligodendrocytes are sensitive to reactive oxygen species and oxidative stress [7,8]. They have been shown to participate intricately in immune mediated processes by producing immune regulatory factors and expressing receptors to communicate with microglia [9]. As it becomes more apparent that astrocytes participate in immune mediated processes as well, their cross-talk with oligodendrocytes might elucidate new mechanisms in neuroinflammatory diseases. 

The importance of astrocytes in oligodendrocyte functioning is exemplified in primary astrocytopathies such as Alexander disease (AxD) and vanishing white matter (VWM) [10] where astrocyte damage leads to demyelination and oligodendrocyte death. In osmotic demyelination syndrome astrocyte death is observed due to loss of gap junctions and proteostasis defects in astrocytes prior to oligodendrocyte loss and demyelination [11,12,13]. In addition, astrocyte dysfunction has been associated with many other neurological diseases including epilepsy [14], amyotrophic lateral sclerosis (ALS) [15], Huntington’s disease (HD) [16], and Alzheimer’s disease (AD) [17]. In neuroinflammatory diseases, such as multiple sclerosis (MS) oligodendrocyte loss might be a consequence of aberrant immune responses. MS is characterized by inflammatory lesions with demyelination, neurodegeneration, and astrogliosis, in which astrocytes and oligodendrocytes are damaged [18,19]. Similarly, numerous other white matter disorders also show important cross-talk between astrocytes and oligodendrocytes (Table 1) [10].

Here we review the evidence for cross-talk between astrocytes and oligodendrocytes demonstrating an emerging role for astrocytes in oligodendrocyte damage, as well as contributing to tissue regeneration and remyelination. Understanding how astrocytes interact with oligodendrocytes will provide a deeper insight into the pathophysiology of neurological disorders that may elucidate new pathways to drug strategies for myelin damage in CNS diseases.

## 2. Astrocyte and Oligodendrocyte Cross-Talk during Brain Development

In neurogenesis, a “gliogenic switch” occurs and dividing neural stem cells develop into glial cells [54,55]. From these cells, both astrocyte precursor cells and oligodendrocyte progenitor cells (OPCs) arise [55,56]. Astrogenesis is mediated through cardiotrophin-1 (CT-1), a factor secreted by cortical neurons. CT-1 induces glial fibrillary acidic protein (GFAP) expression by immature astrocytes through activation of the janus kinase signal transducer and activator of transcription proteins (JAK-STAT). The importance of CT-1 is exemplified by the 50–80% decrease in GFAP expression in CT-1 knock-out mice [57]. Astrogenesis-related genes are silenced during the neurogenic period through epigenetic mechanisms [57,58,59]. Oligodendrogenesis, on the other hand, is subject to a morphogen gradient of Sonic hedgehog (Shh) and bone morphogenic protein (BMP) and OPCs arise on the ventral side of the neural tube [60,61]. Critical in proliferation and timing of oligodendrocyte maturation is secretion of platelet derived growth factor AA (PDGF-AA) by astrocytes [62]. Once generated, OPCs migrate due to chemokines and Shh signalling, all while being guided by astrocytes [63]. In the optic nerve, astrocytes transiently express high levels of the megalin receptor that regulates the availability of Shh in the microenvironment and thus guides OPC migration. Inhibition of the megalin receptor has been shown to result in impaired migration of OPCs to the optic nerve [64]. Furthermore, astrocytes tightly control release of BMPs and prevent maturation of OPCs into myelin-producing oligodendrocytes [65]. Clearly, cross-talk between astrocytes and oligodendrocytes during development is essential for migration and maturation of OPCs through the CNS. 

Various areas in the brain give rise to different types of astrocytes. Fibrous astrocytes are located in the white matter while protoplasmic astrocytes are present in the grey matter. These phenotypes of astrocytes differ in morphology and expression patterns. One example is the expression of excitatory amino acid transporters (EAATs), which is higher in the white matter and results in extracellular glutamate levels being lower in white than in grey matter [3,5,66,67]. Additionally, the astrocytic syncytium formed by protoplasmic astrocytes is larger than that of fibrous astrocytes [67]. The differences observed in morphology and protein expression impact the way these cells interact with their environment and with other glia cells such as oligodendrocytes.

During development astrocytes provide critical metabolic support of oligodendrocytes by supplying e.g., sterol regulatory element-binding protein (SREBP) cleavage-activating protein, a protein essential in lipid production. Mice in which SREBP cleavage activating protein is conditionally knocked out in astrocytes, develop microcephaly and a decrease in white matter volume [68], indicating the importance of astrocyte-derived lipids in myelination. Astrocytes also provide cholesterol for myelin production, and since cholesterol cannot cross the BBB it has to be synthesized de novo in the CNS by astrocytes and oligodendrocytes [3,69,70]. However, inhibition of the oligodendrocyte cholesterol synthesis pathway in mice leads to a delay in myelination suggesting cholesterol production by oligodendrocytes and astrocytes is critical for early myelination [70]. This suggests that cholesterol availability is a rate-limiting factor in myelin production. In experimental autoimmune encephalomyelitis (EAE), an animal model of MS, the cholesterol synthesis pathway is downregulated in astrocytes of the cerebellum and spinal cord [69]. Determining whether this is a cause of limited remyelination requires more investigation. The metabolite exchange between oligodendrocytes and astrocytes may be key for astrocytic leukodystrophies, as disturbed astrocyte function in these disorders may limit lipid exchange from astrocytes to oligodendrocytes. 

## 3. Astrocytic Communication with Oligodendrocytes

### 3.1. Blood-Brain Barrier Interactions

Astrocyte end-feet cover up to 90% of the brain vasculature and are exchange sites for nutrients, metabolites, and ions from the blood to the brain. BBB dysfunction is a key step in the pathogenesis of inflammatory and neurodegenerative CNS diseases [71]. 

Iron from the blood is provided by astrocytes to oligodendrocytes through endocytosis and transferred to the cells as protein-bound iron. Iron is essential for several enzymatic functions of oligodendrocytes, such as energy metabolism enzymes, including the mitochondrial respiratory chain protein complexes I-IV, which use it as a co-factor [4,72]. When oligodendrocytes are deprived of iron, proliferation and differentiation of OPCs is impaired as shown in vitro, leading to a delay in remyelination after injury in vivo [72,73]. The importance of iron in myelination is exemplified by prenatal iron deficiency in which abnormal oligodendrocyte distribution is observed [73]. Abnormalities in iron metabolism are also reported in MS [72] and HD [74] and restoration of normal metabolism is required for remyelination. Maintenance and development of the BBB is regulated by astrocytic Shh [75]. In MS, Shh acts as an anti-inflammatory molecule at the level of the neurovascular unit and is increased during neuroinflammation to promote BBB repair and integrity [75]. These examples underscore the critical role of astrocytes in BBB functioning in order to provide metabolic support to oligodendrocytes, essential in processes such as myelination. 

While astrocytes are considered to be key players in maintaining BBB integrity, OPCs have also been shown to play a role in BBB integrity through TGF-β signalling [76]. Additionally, BBB integrity is enhanced by OPCs through PDGF-BB/PDGFRα signalling while oligodendrocytes control BBB integrity independent of this pathway [77]. Conversely, a recent study combining pathology, in vivo and in vitro cultures indicates that clusters of OPCs contribute to altered vascular permeability by impacting the astrocyte foot processes in MS [78]. OPCs require a vascular scaffold for migration throughout the CNS to repopulate demyelinated areas in MS but detachment of the vasculature fails which results in a disruption of the BBB integrity [78].

### 3.2. Gap Junctions Connect Astrocytes and Oligodendrocytes

Astrocytes are connected to other glial cells via gap junctions, allowing free flow of ions and small metabolites. Gap junctions between astrocytes are made up of connexin (Cx) 30 and/or 43 that forms either homotypic (Cx30:Cx30 or Cx43:Cx43) or heterotypic channels (Cx30:Cx43). Using these gap junctions, astrocytes form a syncytium with free flow of small molecules including gliotransmitters and lactate that aids buffering of K^+^ [20,79]. Astrocytes express Cx30 and Cx43 that couples to adjacent oligodendrocytes expressing Cx32 and Cx47 by forming heterotypic gap junctions respectively Cx30:Cx32 and Cx43:Cx47 [6,79]. This physical contact is important in oligodendrocyte maturation and is often disrupted in demyelinating conditions. In EAE the reduction in Cx47 and Cx32 reduces oligodendrocyte-oligodendrocyte and astrocyte-oligodendrocyte interactions [20]. This reduction is also observed in active and chronic lesions in MS, neuromyelitis optica (NMO) and Baló’s disease [80]. Absence of Cx47 or Cx32 in oligodendrocytes exacerbates clinical EAE in mice associated with increased myelin loss but does not affect Cx30 and Cx43 expression in astrocytes [81]. Pathogenic mutations in Cx32 also contribute to Charcot-Marie-Tooth disease characterized by peripheral demyelination and neuropathy [80]. In contrast, Cx43 is upregulated in remyelinating MS lesions, emphasizing the importance of communication via gap junctions in remyelination [80]. The detrimental effect of Cx loss on remyelination may be attributed to the necessity of trophic support of oligodendrocytes by astrocytes, although whether the loss of Cx in gap junctions is the cause or consequence of myelin damage is unclear [82].

## 4. Astrocytes and Oligodendrocytes Play Active Roles in Immune Responses

**Figure 1:**
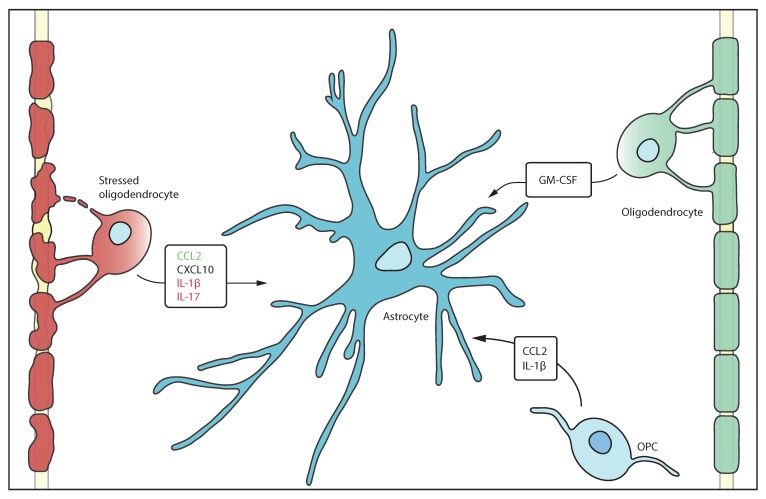
Oligodendrocytes secrete factors that impact on astrocytes. Stressed oligodendrocytes release factors that have beneficial effects (green) on astrocytes such as CCL2 to reduce inflammation. In contrast detrimental factors (red) such as IL-1β exacerbates inflammation. Healthy oligodendrocytes and OPCs also interact with astrocytes by secretion of GM-CSF and CCL2 as well as IL-1β.

Emerging studies have changed the perception that astrocytes and oligodendrocytes are solely bystanders in inflammatory processes. In infectious and inflammatory CNS diseases oligodendrocytes have been reported to act as antigen presenting cells and produce immune molecules [9] (Table 2). In neuroinflammation oligodendrocytes express many factors known to activate astrocytes [83,84] (Figure 1). For example, in vitro astrocytes express receptors for e.g., CCL2 and CXCL10 which are mostly secreted to attract monocytes and macrophages [85,86,87,88]. In MS lesions oligodendrocyte and astrocyte expression of IL-17 suggests that glia, as well as T cells, promote the pro-inflammatory environment that attracts macrophages to the lesion [89]. In mice, administration of cuprizone, that damages and ablates oligodendrocytes, both oligodendrocytes and OPCs secrete IL-1β, a known pro-inflammatory cytokine [90,91,92]. CXCL1, CXCL2, CXCL3, CXCL5, and CXCL6 all bind the CXCR2 receptor, which is constitutively expressed on oligodendrocytes, but not present on astrocytes [88,93]. The CXCR2 receptor is upregulated in response to these cytokines that are secreted by oligodendrocytes, supporting autocrine regulation. Several CXCR2 ligands have previously been associated with OPC proliferation and differentiation [94], indicating that oligodendrocytes regulate their own proliferation. Granulocyte macrophage colony stimulating factor (GM-CSF) is upregulated in resting oligodendrocytes [93] which has been found to be anti-apoptotic for neurons and neuroprotective in models of stroke.

Additionally, astrocytes secrete CXCL1 in spinal cord injury and in MS lesions, both in vivo and in vitro, which may act to recruit oligodendrocytes [88,95]. Gap junctions are also reported to play an immunoregulatory role for example Cx43 loss in astrocytes increases recruitment of immune cells in the brain as well as inducing an atypical reactive astrocyte phenotype that secretes both pro- and anti-inflammatory factors [82,96,97].

**Figure 2:**
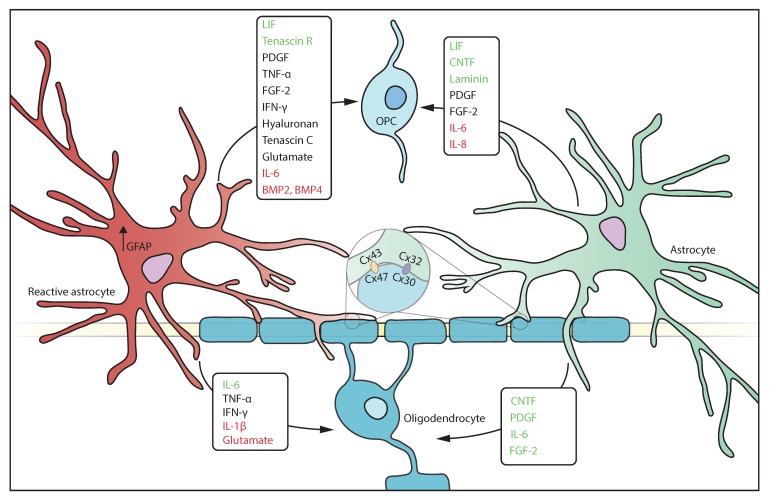
Astrocytes release a wide variety of molecules that impact oligodendrocyte functioning. Reactive and homeostatic astrocytes can release both beneficial (green) as well as detrimental (red) molecules. Most molecules that are secreted by astrocytes have a context dependent effect as well as a differential effect on oligodendrocytes and OPCs.

Many immune factors are secreted by both oligodendrocytes and astrocytes in vitro i.e., IL-1β, CXCL10 and IL17, underscoring a possible immune function of these cells [90]. In addition, astrocytes also secrete tumour necrosis factor-α (TNF-α), IL-1β, interferon-γ (IFN-γ), fibroblast growth factor-2 (FGF-2), PDGF, and BMPs, factors known to influence oligodendrocytes and OPCs [3,98] (Figure 2, Table 2). TNF-α is recognized by TNFR1 and induces pro-inflammatory effects, while binding to TNF-αR2 induces anti-inflammatory effects. Both TNFR1 and TNFR2 are expressed on oligodendrocytes [9], and both are upregulated during inflammation [8] indicating that oligodendrocytes could trigger both pro- and anti-inflammatory responses. Likewise, astrocytes express predominantly TNFR1 but are capable of upregulating TNFR2 after stimulation by TNF-α [3,99], suggesting an autocrine feedback loop. While inhibition of TNF-α is an effective therapy in autoimmune diseases such as rheumatoid arthritis, this approach has been less straightforward in MS [100], recent data shows that selective modulation of TNFRs by activating TNFR2 and/or silencing TNFR1 might have therapeutic potential [101]. IL-1β is expressed by astrocytes during ischemic stroke, as well as neuroinflammatory disease although the precise mechanisms of IL-1β remain unclear [102,103]. IL-1β was also found in active MS lesions in reactive astrocytes and in pre-active lesions where it might act on oligodendrocytes and astrocytes in lesion formation [104]. IFN-γ has both pro- and anti-inflammatory effects, as treatment with IFN-γ exacerbates MS pathology, but also induces neurotrophic factor production in astrocytes, which are also able to produce IFN-γ [105,106]. FGF-2 is secreted by astrocytes after focal demyelination in mice, and has been shown to promote OPC proliferation yet inhibit their differentiation to oligodendrocytes [4,21]. BMPs are upregulated in EAE, and direct OPC differentiation into the astrocyte lineage [21]. Lastly, insulin-like growth factor-1 (IGF-1) also induces OPC maturation [107]. 

## 5. Astrocyte—Oligodendrocyte Interplay in Disease

### 5.1. Reactive Gliosis and Glial Scar Formation

Reactive astrocytes are a hallmark of many CNS diseases for example in MS lesions [3,22,25,126], around the injured site during spinal cord injury (SCI) [41], within Rosenthal fibres in AxD [127], after ischemic stroke [128], and near amyloid plaques in AD [129], indicating their importance in both classic white matter and grey matter disease. Reactive gliosis is a spectrum rather than an all-or-nothing reaction, and the severity may differ between diseases, patients, or even within a patient. Mildly reactive astrocytes are associated with milder CNS injury or inflammation, and do not proliferate, showing only moderate changes in gene expression. Severely reactive astrocytes are characterized by upregulation of GFAP, hypertrophy and proliferation, and are present in severe injury and infection, as well as in chronic neurodegenerative disease. The most severe reaction is the glial scar, where astrocytes proliferate and intertwine to form a physical barrier that surrounds injured CNS tissue and isolates it from healthy tissue. It is associated with severe necrosis or inflammation [130]. 

In the acute stages of CNS damage, glial scarring is essential to prevent more widespread inflammation and the spread of toxic factors, protecting neurons from secondary degeneration [131]. An astrocyte-specific STAT3 knock-out inhibits formation of the glial scar, and leads to increased inflammation and motor dysfunction in mice after SCI [2,4,98]. On the other hand, in an AD mouse model, inhibition of the JAK2-STAT3 pathway leads to reduced astrocyte reactivity and increased learning abilities [132]. Glial scars are also involved in restoration of BBB integrity in inflammatory CNS disorders [20,131]. However, in the chronic stages, the glial scars inhibit OPC migration and differentiation and is thus considered to be detrimental blocking tissue repair [3,4,66,103]. This is observed in ischemic stroke, where the glial scar secretes growth inhibiting factors that prevent axonal regrowth [128], and in MS, where OPC migration into demyelinated lesions is inhibited [22]. 

Astrocytes become reactive in response to both direct and indirect activation; indirect activation is mediated by cytokines secreted by microglia, while direct activation is mediated by damage or pathogen associated molecular patterns that are released by pathogens or during cell death, oxidative stress, or chemical stress [3,22]. This implies that oligodendrocyte injury induces astrocyte reactivity. Upon activation, astrocytes secrete factors e.g., TNF-α, IL-1β, IL-6, brain derived neurotrophic factor (BDNF), leukemia inhibitory factor (LIF), CCL2, and CXCL10 [3,22,98,103,133]. These factors play a critical role in generating the immune responses during infection or damage, but also lead to collateral damage of oligodendrocytes and OPCs. The glial scar is essential to keep these factors isolated in the acute phase of disease, and abolishing it is adverse to recovery, while modulation of the glial scar in the chronic phase of disease may stimulate remyelination in white matter disorders.

### 5.2. Astrocytes in Neuroinflammation 

The NF-κB pathway is a major inflammatory pathway involved in activation of the innate and adaptive immune responses essential for e.g., generation of T-cell and B-cells. The pathway is constitutively active in many inflammatory disorders of the white matter [90,134]. In vitro, astrocytes upregulate NF-κB in response to pro-inflammatory cytokines such as IL-17, IL-1β, and TNF-α [90,135]. In vivo, overexpression of the NF-κB inhibitor IκBα in astrocytes results in protection of oligodendrocytes via reduced leukocyte infiltration and lower levels of chemokines during EAE [136]. NF-κB is also relevant in other CNS disorders that are not classically seen as white matter disorders, including AD, where amyloid-β plaques induce NF-κB activation in an astrocyte-specific manner [137]. In SOD1 mice, a mouse model of ALS, astrocytic NF-κB promotes degeneration of motor neurons and accelerates disease progression [138]. Subtle white matter changes are found in neurodegenerative diseases as early as pre-clinical AD where the NF-κB pathway could play a role in exacerbating inflammatory signaling [139]. Intervention in this pathway is effective, as demonstrated by the MS drug laquinimod, which inhibits astrocytic NF-κB expression [4,134]. NF-κB signalling represents an important inflammatory pathway in various neurological disorders that is frequently used by astrocytes to exacerbate inflammation. Alleviation of oligodendrocyte pathology via astrocytic NF-κB targeting may be relevant in more white matter disorders, and its use in MS treatment is proof of concept for the relevance of cross-talk in white matter disease therapy. 

### 5.3. Excitotoxicity

Oligodendrocytes are sensitive to excitotoxic damage due to their expression of α-amino-3-hydroxy-5-methyl-4-isoxazolepropionic acid (AMPA) and kainate receptors. In EAE, treatment with AMPA and kainate antagonists significantly reduces oligodendrocyte death and disease severity, suggesting a role for excitotoxic cell death in MS [22,140]. Moreover, TNF-α triggers astrocytic upregulation of prostaglandin-E2 in vitro, which induces release of glutamate into the extracellular space [141], indicating that neuroinflammation exacerbates excitotoxic damage, leading to oligodendrocyte death. Excitotoxicity is further facilitated by downregulation of EAATs, which occurs in the senile plaques in AD, and in ALS [129,137]. Excitotoxicity in oligodendrocytes is not just glutamate-mediated, but also ATP-mediated, via overstimulation of the P2X purinoreceptor-7 (P2X7) ATP receptors. Similar to the AMPA and kainate receptors, the P2X7 receptor is Ca^2+^ permeable, and the intracellular Ca^2+^ damages oligodendrocytes.

Studies by Matute and colleagues show that in mice P2X7 antagonists prevent ATP toxicity in oligodendrocytes [7]. P2X7 receptors are significantly increased in oligodendrocytes in the optic nerves of people with MS compared to healthy controls, indicating that ATP toxicity might be a relevant pathogenic mechanism in disease [142]. ATP toxicity is also pathogenic after SCI, increasing demyelination and neuronal death after injury [143]. In support of this, treatment of rats with P2X7 antagonists increases neuronal survival and functional recovery after SCI [143]. Stimulation of the P2X7 receptor of neonatal rat-derived astrocytes results in glutamate release, supplying the environment with more excitotoxic molecules [142]. Astrocytic overexpression of P2X7 was also found in the white and grey matter in secondary progressive MS [144] as well as upon stimulation with IL-1β [123], suggesting that this signalling pathway is especially relevant during inflammation. 

P2X7 is also of importance in epilepsy, since sufferers have higher P2X7 expression than healthy controls in the neocortical nerve terminals. P2X7 antagonist treatment decreases the severity and number of epileptic seizures in rats [145]. Excitotoxicity is a relevant mechanism of cell death in many disorders, and astrocyte-oligodendrocyte cross-talk plays an important role here, as astrocytes are able to create a hostile environment with glutamate and ATP which then damages oligodendrocytes. Inhibition of astrocytic glutamate release or increasing the activity of the EAAT receptors may thus be a relevant treatment mechanism in epilepsy or white matter diseases.

## 6. Astrocyte Control of Remyelination and the Extracellular Matrix

The white matter of the brain primarily consists of myelinated axons formed by oligodendrocytes, after differentiating from OPCs [4]. In demyelinating diseases such as MS and NMO, functional recovery requires remyelination. Although in these diseases astrocytes are known to be detrimental to oligodendrocytes and OPCs, they also promote and mediate remyelination [7,41]. For example, in vivo ablation of astrocytes results in impaired recovery from SCI [2]. In MS or SCI remyelination occurs but often fails despite the presence of significant numbers of OPCs suggesting the lack of remyelination is likely due to a failure in OPC differentiation rather than migration [20,146,147]. However, migration failure and clustering of OPCs at astrocyte endfeet indicates that astrocytes may also play a role in restricting migration of OPCs [78]. Recruitment of OPCs to the demyelinating area occurs through astrocyte chemokine signalling of IL-1β and CCL2, confirming the necessity of cellular cross-talk in remyelination [20,148]. After migration, OPCs exit the cell cycle and differentiate into oligodendrocytes through stimulation of PDGF and FGF-2 [147]. FGF-2 is highly upregulated by astrocytes in remyelinating spinal cord lesions where it acts on oligodendrocytes as well as in autocrine fashion on astrocytes [149]. Recently, a new study has found that OPCs might not be as important for remyelination as previously thought. Remyelination was found to be mainly dependent on the pool of surviving mature oligodendrocytes present in the lesions based on carbon dating of oligodendrocytes [150].

Astrocytes influence oligodendrocytes via modification of the extracellular matrix (ECM). A major ECM component secreted by astrocytes is hyaluronan, which acts on T-cells and OPCs, blocking OPC differentiation into oligodendrocytes and promoting astrocytic differentiation [41]. Hyaluronan is especially abundant in white matter lesions of MS patients [21], as well as patients with rare familial leukodystrophies VWM [50] and AxD [127]. Exaggerated hyaluronan secretion is a common feature of leukodystrophies, and likely has a role in neurological pathogenesis. Another astrocytic ECM factor is laminin that controls the differentiation and migration of OPCs, and promotes their survival by binding integrin and dystroglycan receptors. Mutations in laminin result in profound muscular and white matter abnormalities [151,152,153]. In inflammatory conditions, reactive astrocytes also produce tenascin C and R. Tenascin C is linked to inhibition of OPC migration, but tenascin R induces myelin gene expression and OPC differentiation. In chronic MS plaques, both tenascin C and R were shown to be upregulated in reactive astrocytes [153]. Lastly, astrocytes secrete proteoglycans that inhibit remyelination in high concentrations [154]. Proteoglycans also capture chemokines and growth factors, localizing them and targeting immune cells to the area of inflammation. This helps to prevent immune-mediated collateral damage [131]. These studies underscore the importance of the ECM in providing a healthy environment for remyelination. If disrupted, a remyelination promoting environment turns inhibitory, leading to impaired differentiation and proliferation of OPCs. Astrocytes are an important source of many ECM factors, and communicate with and influence OPCs and oligodendrocytes via secretion of ECM factors.

In inflammatory CNS conditions infiltration of immune cells is a hallmark of disease and heavily dependent on the breakdown of the ECM. MMP2 and 9 are important in degradation of the *Lamina basalis*, as well as infiltration of immune cells into the brain parenchyma. The activity of these proteins is regulated by tissue inhibitors of metalloproteinases (TIMPs). Although astrocytes express MMP2 and 9 both in vivo and in vitro, they also produce TIMP-1 [103]. Astrocytes promote oligodendrogenesis during and after injury through secretion of BDNF and TIMP-1 [155,156]. This is also shown in TIMP-1 deficient mice that exhibit defective myelin repair [20], indicating the importance of the ECM in remyelination. Another MMP that is active in remyelination is MMP7, which cleaves fibronectin aggregates present in demyelinating lesions in MS. These aggregates prevent OPC maturation and remyelination. Secreted proMMP7 is activated by astrocytic MMP3, indicating that astrocytes assist in this cleavage [157]. This shows that astrocytes are not only involved in the building of the ECM, but also in its breakdown and maintenance.

In summary, astrocytic dysfunction results in a toxic extracellular environment with high levels of excitotoxic molecules and pro-inflammatory cytokines such as IL-1β and TNF-α. On the other hand, their basal functions are essential in maintaining a healthy brain microenvironment where oligodendrocytes thrive and remyelinate the CNS. Although astrocytes can be detrimental in neurological disease they are also essential for the recovery from damage. Astrocytes are particularly important in early recovery by supporting oligodendrocyte migration and OPC differentiation. However, astrocytes become pathological in the chronic phase, exemplified by the glial scar formation in SCI or MS, in which hypertrophic astrocytes produce many factors that induce a harmful environment for mature oligodendrocytes and inhibit OPC differentiation [41,131]. 

## 7. Conclusions

Astrocyte and oligodendrocyte interactions in healthy conditions and disease are complex and multifaceted. The widely considered view that astrocytes only react to damage in neurological diseases is changing to embrace the emerging evidence that these cells are essential to the development of the healthy CNS. On the other hand, astrocytes are involved in the pathogenesis of several CNS diseases since loss of normal trophic functions of astrocytes results in damage to neurons and oligodendrocytes thereby exacerbating pathology. 

Furthermore, astrocytes are important for the regenerative capacities of the brain aiding oligodendrocyte proliferation, maturation and migration—a key step in repair in diseases such as MS and other demyelinating diseases. There is also a growing awareness that astrocytes and oligodendrocytes are not only targets for autoimmune responses in the context of neuroinflammation. Astrocytes play an important role as innate immune cells, e.g., by secreting chemokines, and as such influence other glia cells. 

Future studies into the communication between astrocytes and oligodendrocytes as well as their impact on other CNS cell types will provide new clues for controlling innate immunity and aiding repair in the CNS.

## Figures and Tables

**Table 1:** Astrocyte involvement in white matter CNS diseases ^1^.

	Disease	Pathology	Detrimental Impact on Astrocytes	Beneficial Impact on Astrocytes	References
Inflammatory	MS	Inflammation, myelin loss, neurodegeneration, astrogliosis, astrocyte damage.	BBB damage, impaired signal transduction and glutamate clearance. Reduced OPC proliferation	Gliosis may aid remyelination and regenerate integrity of BBB, aid remyelination and provide trophic support	[5,20,21,22,23]
	NMO	Inflammation, myelin loss in optic nerve and spinal cord. Reduction in AQP4 and GFAP. Decreased EAAT2.	Impaired water and ion homeostasis, impaired glutamate clearance	Stimulation of remyelination, trophic support	[21,24,25,26]
	ADEM	Widespread CNS inflammation associated with infection.	Dependent on infectious agent	Infection may trigger protective response via TLR-dependent mechanism	[27]
	AHL	Perivascular demyelination, inflammation, oedema, haemorrhages. Hyper-reactive astrocytes.	Swelling of protoplasmic and fibrous astrocyte end-feet, beading consistent with degeneration.	Demyelination is secondary to astrocyte injury indicating a beneficial effect of astrocytes in early disease	[28]
Infectious	PML	Cytolytic JC virus induces oligodendrocytes death and focal myelin loss. Abnormal astrocytes with inclusion bodies.	Astrocytes aid the spread of JC virus to neighbouring oligodendrocytes	Unknown	[29,30,31]
	SSPE	Viral inclusion bodies in neurons, neuronal damage and loss. Virion inclusion in some astrocytes.	Infection of (perivascular) astrocytes may aid spread of virus	Reactive gliosis in longstanding disease may be beneficial	[32,33]
	Congenital CMV	Encephalitis, microglial activation.	CMV infection of astrocytes induces TGF-beta known to enhance productive infection. Infection of foetal astrocytes alters uptake and metabolism of glutamate	Unknown	[34,35]
Toxic-Metabolic	PNND	Depends on position and type of tumour.	Pathogenic antibodies and CD8+ T cells to astrocytic antigens expressed on tumour induces neurological damage	Unknown	[36,37]
Hypoxia-Ischemia	Binswanger disease	Chronic microvascular leukoencephalopathy, white matter lesions, axonal damage.	Damage to BBB leads to peri-infarct reactive astrocytes	Unknown	[38]
	Cerebral hypoxia and ischemia in new-borns	Diffuse white matter damage, gliosis, decrease in oligodendrocytes.	Reactive astrocytes form a glia scar and secret inflammatory molecules e.g., ROS	Astrocytes produce PDGF, IGF-1, elevated levels of EAAT2 aid glutamate removal in response to hypoxia. VEGF production mobilises stem cells. BDNF reduces apoptosis.	[39,40]
TBI	Diffuse axonal injury	Axonal damage, tau accumulation, secondary white matter damage, astrogliosis.	Glial scar inhibits remyelination and axonal regrowth	Glial scar prevents spread of toxic molecules	[2,41]
Lysosomal Storage	MLD	Accumulated sulfatides leads to demyelination, sparing of U-fibres. Eosinophilic granules in macrophages, metachromasia.	Sulfatide accumulates in astrocytes impairing differentiation	Unknown	[42]
Peroxisomal	X-linked ALD	Defective ABCD1 transport protein. Increased saturated VLCFA in serum. Progressive demyelination. VLCFA accumulate in glia.	Astrocyte stress prior to myelin damage due to accumulated VLCFA. Astrocytes produce ROS and have impaired oxidative ATP synthesis and decreased Ca^2+^ uptake capacity	Unknown	[43,44]
Mitochondrial	Leber’s hereditary optic neuropathy	Loss of retinal ganglion cells, optic nerve degeneration.	Unknown	Unknown	
DNA Repair Defects	Cockayne syndrome	Patchy myelin loss, neuronal loss, astrocytic gliosis, microglia nodules.	Multinucleated astrocytes	Unknown	[45]
Defects in Myelin Genes	PMD	PLP1 duplication or gene alterations, dysmyelination, failure to form myelin.	Increased astrocytic activity, astrogliosis.	Unknown	[46]
AA/Organic Acid Metabolism Disorders	Canavan disease	Mutations of aspartoacylase gene diffuse spongiform white matter degeneration, dysmyelination and intramyelinic oedema. Hypertrophy and hyperplasia of astrocytes.	Metabolic disturbance of mitochondria in abnormal astrocyte	Unknown	[47,48]
Miscellaneous	Alexander disease	Myelin damage, Rosenthal fibres, non-neoplastic astrocytes	Mutations in GFAP lead to diminished glutamate transporter, accumulation of CD44, and loss of EAAT-2. Loss of Cx43 and Cx30	Unknown	[49]
	VWM	Progressive demyelination, blunted dysmorphic astrocytes.	Failure to reach maturity of astrocytes. Overexpression of nestin and GFAPδ	Unknown	[50]
	CADASIL	Diffuse white matter lesions, subcortical infarcts. Granular osmiophilic material in small vessels	Astrocytes undergo autophagy-like cell death. Glia-vascular unit damaged, BBB disturbed	Unknown	[51]
	PMLD	Lack of the gap junction protein Cx47 leads to splitting and decompaction of myelin sheaths and axonal spheroids.	Gap junctions between astrocytes and oligodendrocytes are disturbed compromising oligodendrocyte survival and myelination.	Unknown	[52]

Abbreviations: ADEM, acute disseminated encephalomyelitis; AHL, acute haemorrhagic leukoencephalopathy; AQP4, Aquaporin-4; BBB, blood brain barrier; BDNF, Brain-derived neurotrophic factor; CMV, cytomegalovirus; CNS, central nervous system; EAAT, Excitatory amino acid transporter; GFAP, Glial fibrillary acidic protein; IGF, insulin-like growth factor; MLD, Metachromatic leukodystrophy; MS, multiple sclerosis; NMO, neuromyelitis optica; OPC, oligodendrocyte precursor cell; PDGF, platelet derived growth factor; PMD, Pelizaeus-Merzbacher disease; PMLD, Pelizaeus-Merzbacher-like disease; PML, progressive multifocal leukoencephalopathy; PNND, paraneoplastic neurological disorders; ROS, Reactive oxygen species; SSPE, subacute sclerosing panencephalitis; TBI, traumatic brain injury; TGF, transforming growth factor; TLR, toll-like receptor; VEGF, vascular endothelial growth factor; VLCFA, very long chain fatty acid; VWM, vanishing white matter. ^1^ As classified by van der Knaap and Valk [53].

**Table 2:** Immunologic interplay between astrocytes and oligodendrocytes.

	Detrimental	Beneficial	References
Astrocyte Mediator	Impact on Oligodendrocytes		
TNF-α	Induces demyelination and oligodendrocyte necrosis	Induces PDGF, and LIF on astrocytes which enhances OPC survival and differentiation	[3,108,109,110,111,112]
IL-1β	Induces oligodendrocyte apoptosis and hypomyelination		[102]
IFN-γ	Reversibly reduces OPC proliferation	Limits inflammation, limits Th17 activation, limits IL-1β signalling, protects oligodendrocytes from endoplasmic reticulum stress	[106,113,114,115]
FGF-2	Induces loss of myelin and myelin-producing oligodendrocytes	Induces proliferation of OPCs	[4,116]
BMP	BMPs induce OPC differentiation into the astrocyte lineage		[21,117]
CNTF		Induces proliferation and differentiation of OPCs	[21,105]
IGF-1		Induces OPC differentiation	[21,107,118]
Oligodendrocyte Mediator	Impact on Astrocytes		
CCL2		Reduces IL-6 expression in astrocytes, leading to a less inflammatory environment	[92,119,120]
CXCL10	Induces CXCR3 receptor expression		[119,121]
IL-17	Induces GFAP, IL-1β, and VEGF, reduces BBB integrity Induces astrogliosis		[89,122]
IL-1β	Induces IL-1β and NF-κB, and P2X_7_ receptor.		[90,92,123,124]
GM-CSF		Inhibits glial scar formation. Induces proliferation, and migration of astrocytes	[93,125]

Abbreviations: BMP, bone morphogenic protein, CNTF, ciliary neurotrophic factor; FGF, Fibroblast growth factor; IFN, interferon; IGF, insulin-like growth factor; LIF, leukaemia inhibitory factor; OPC, oligodendrocyte precursor cell; PDGF, Platelet-derived growth factor; TNF, tumour necrosis factor.
